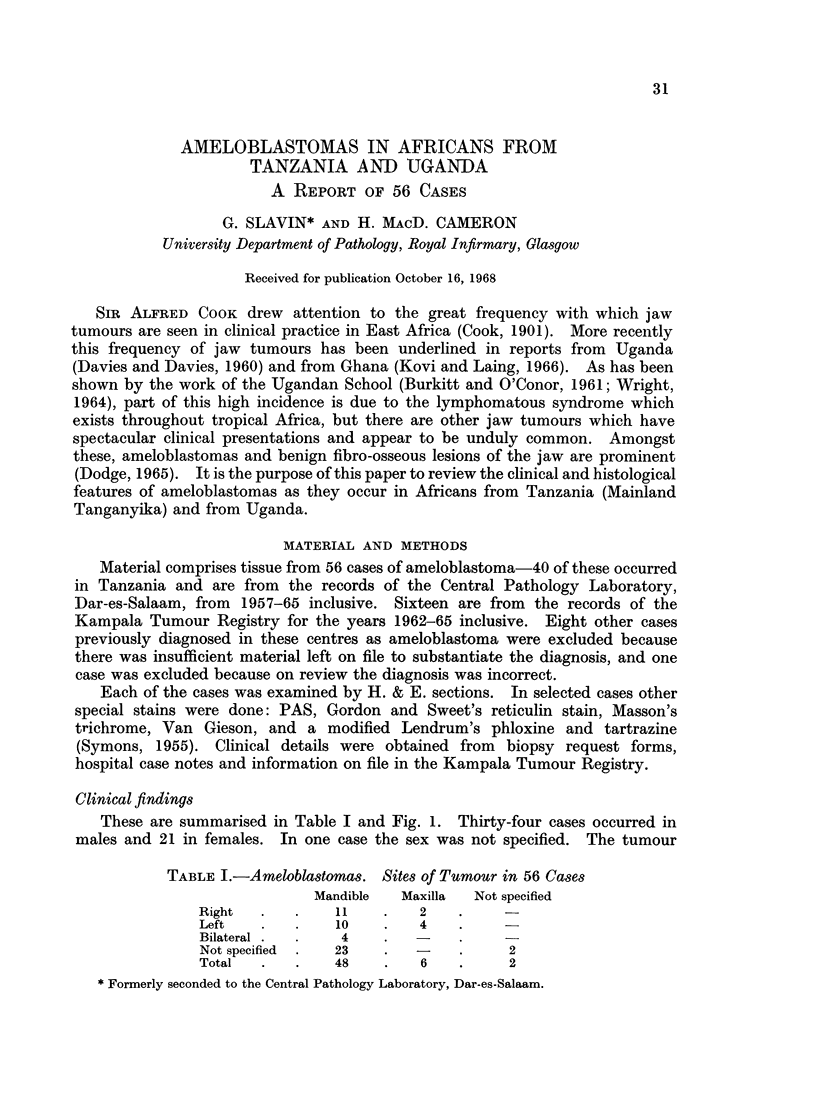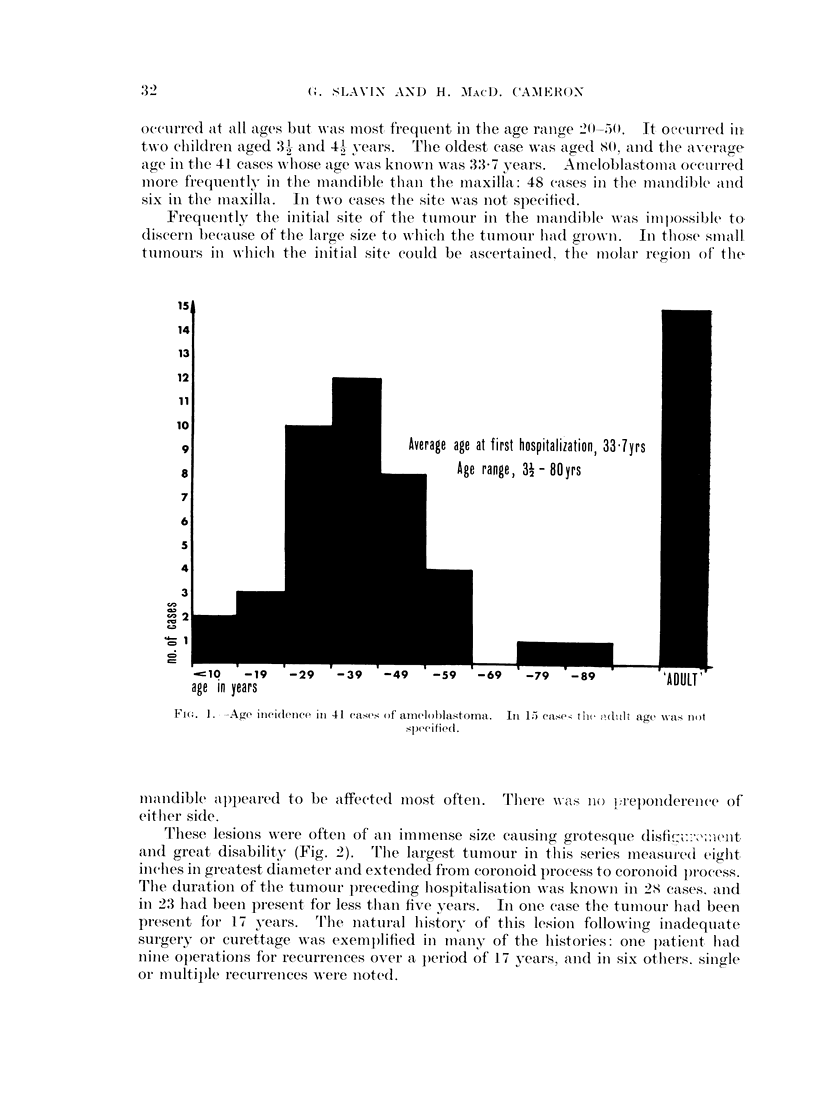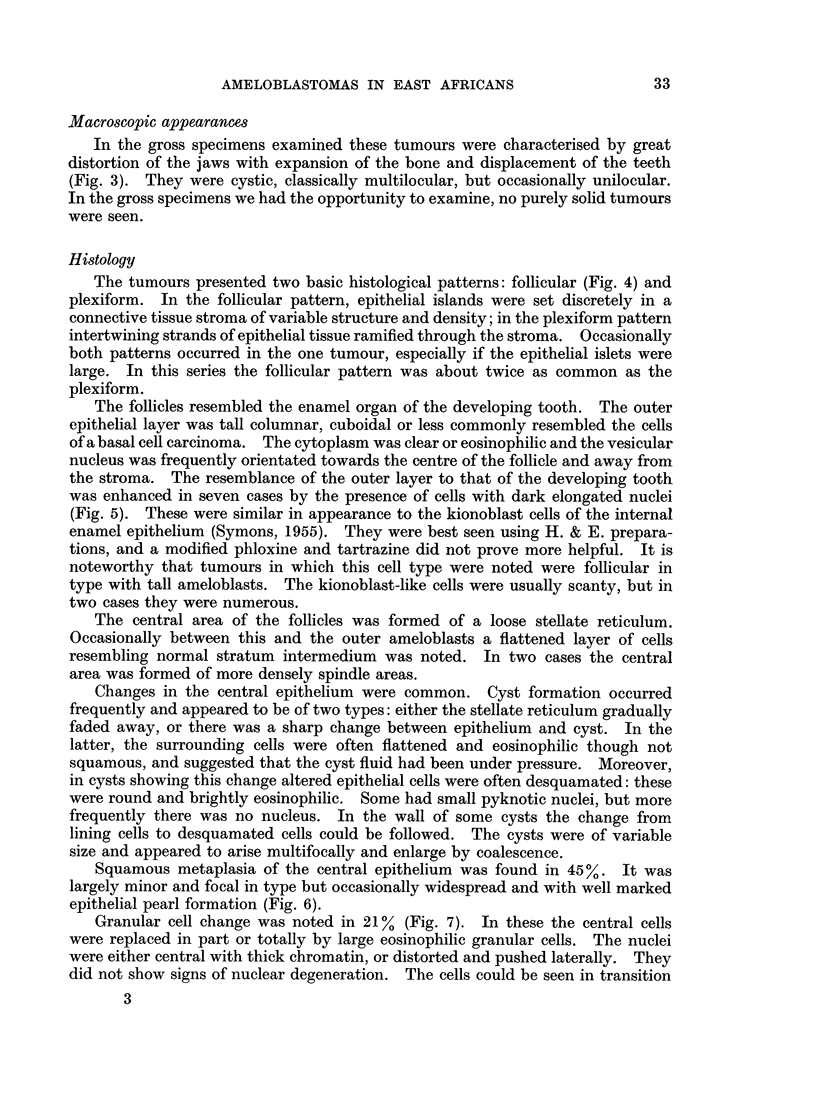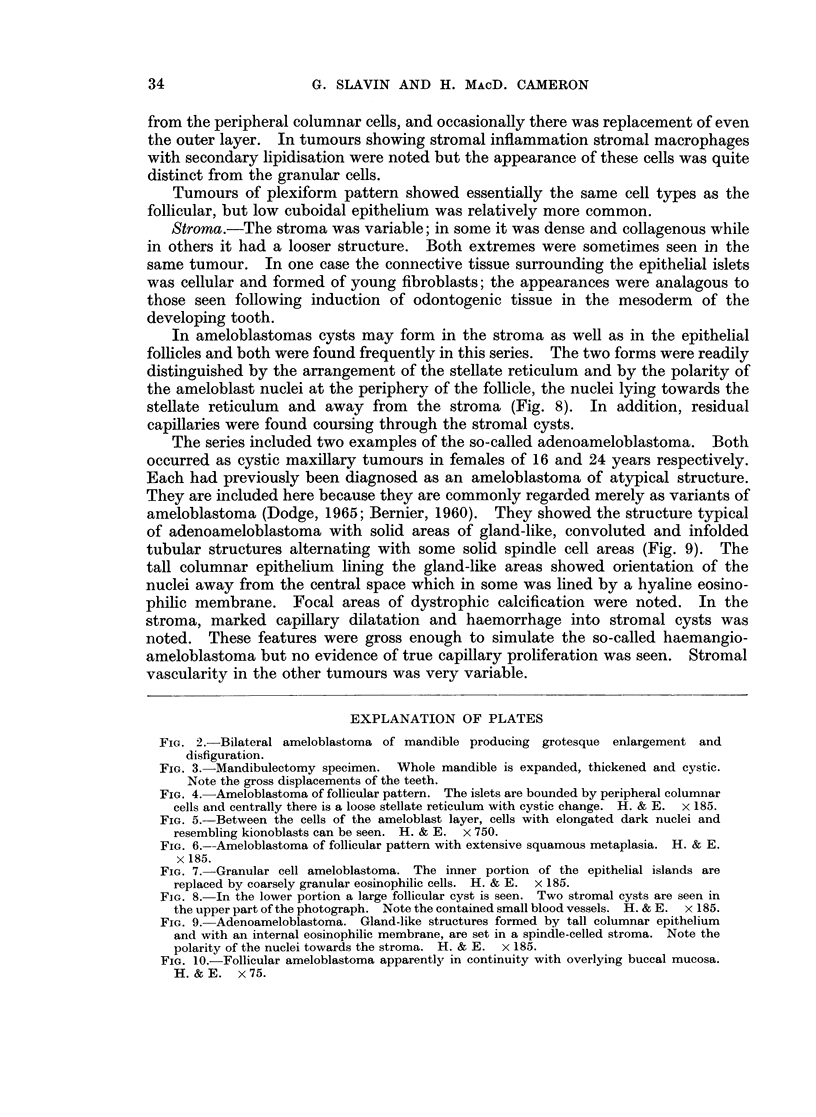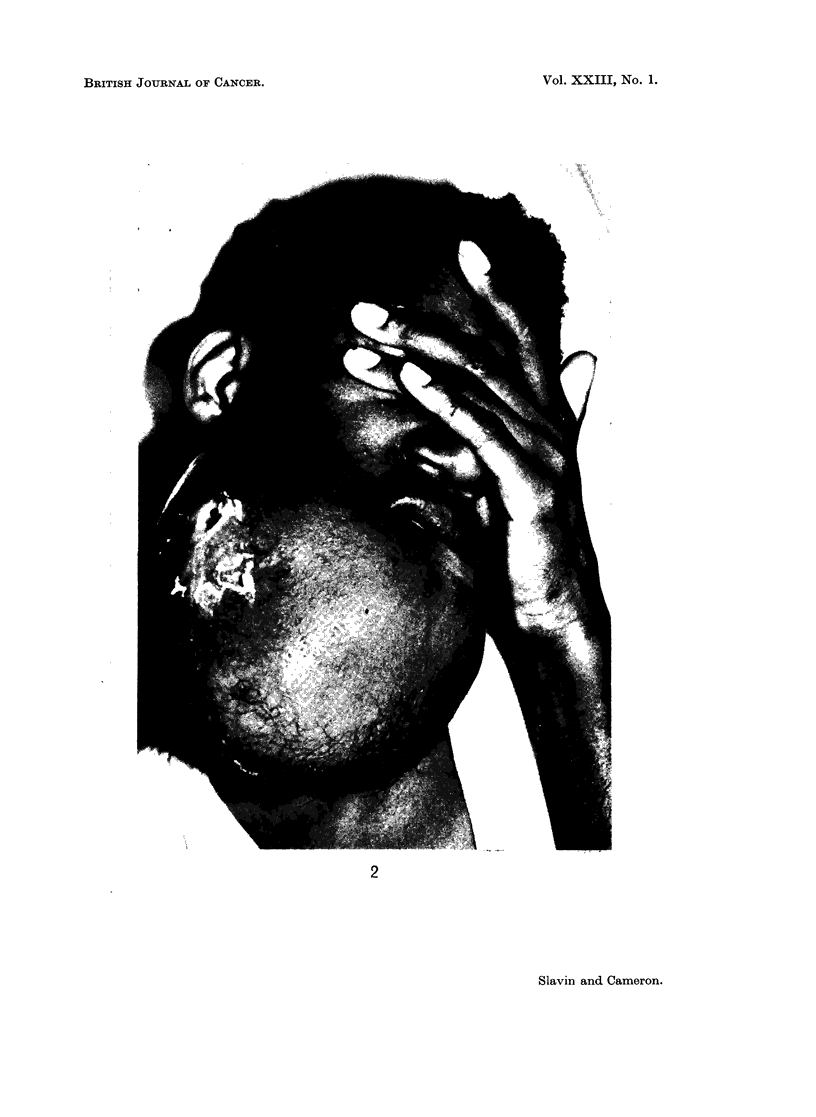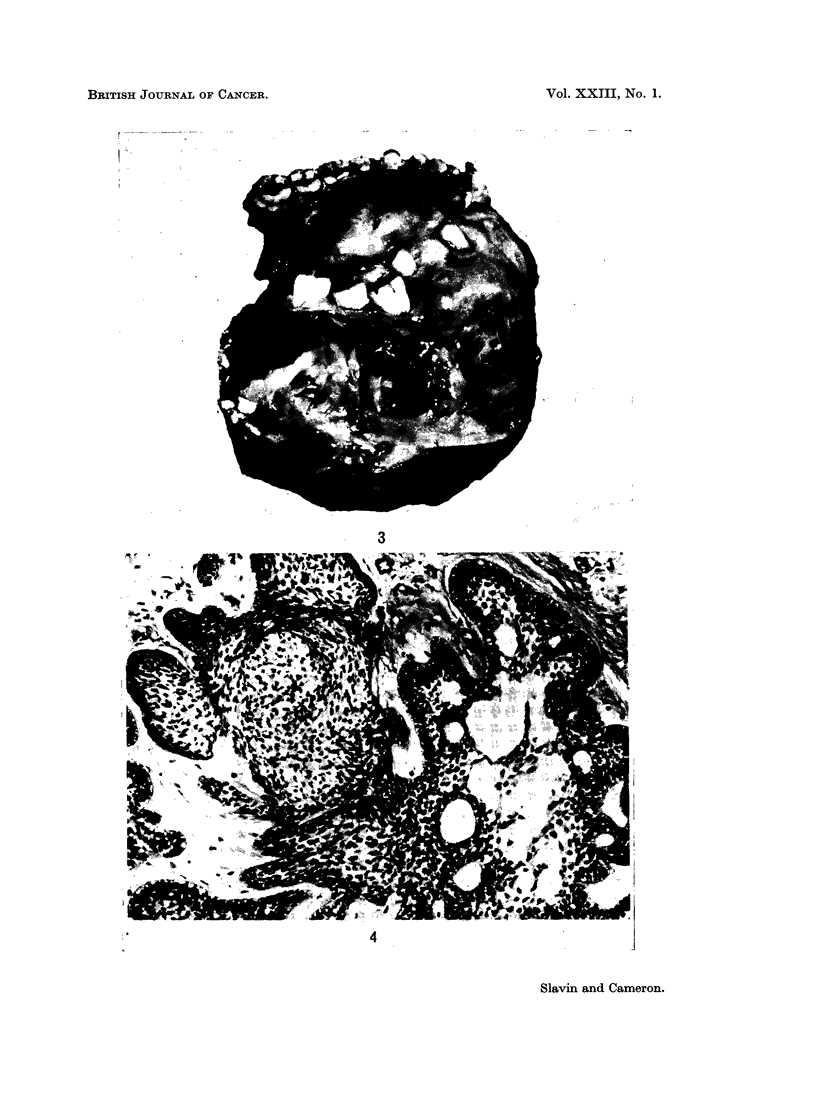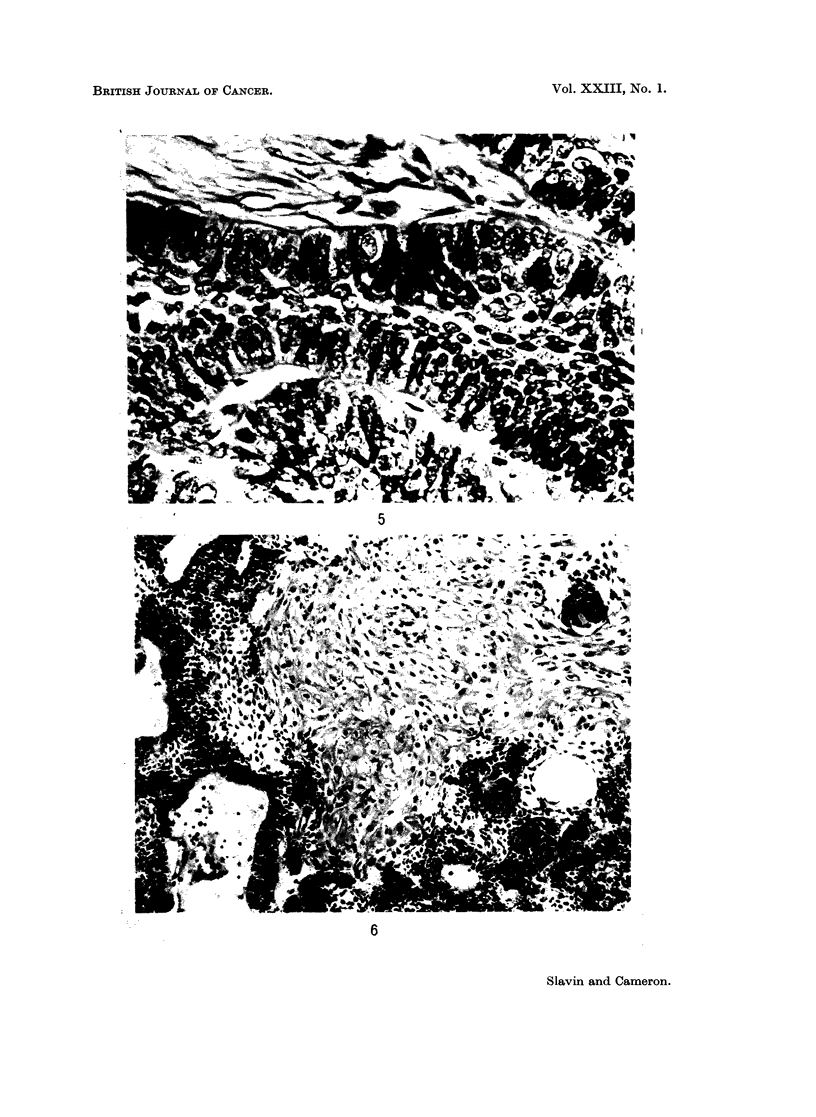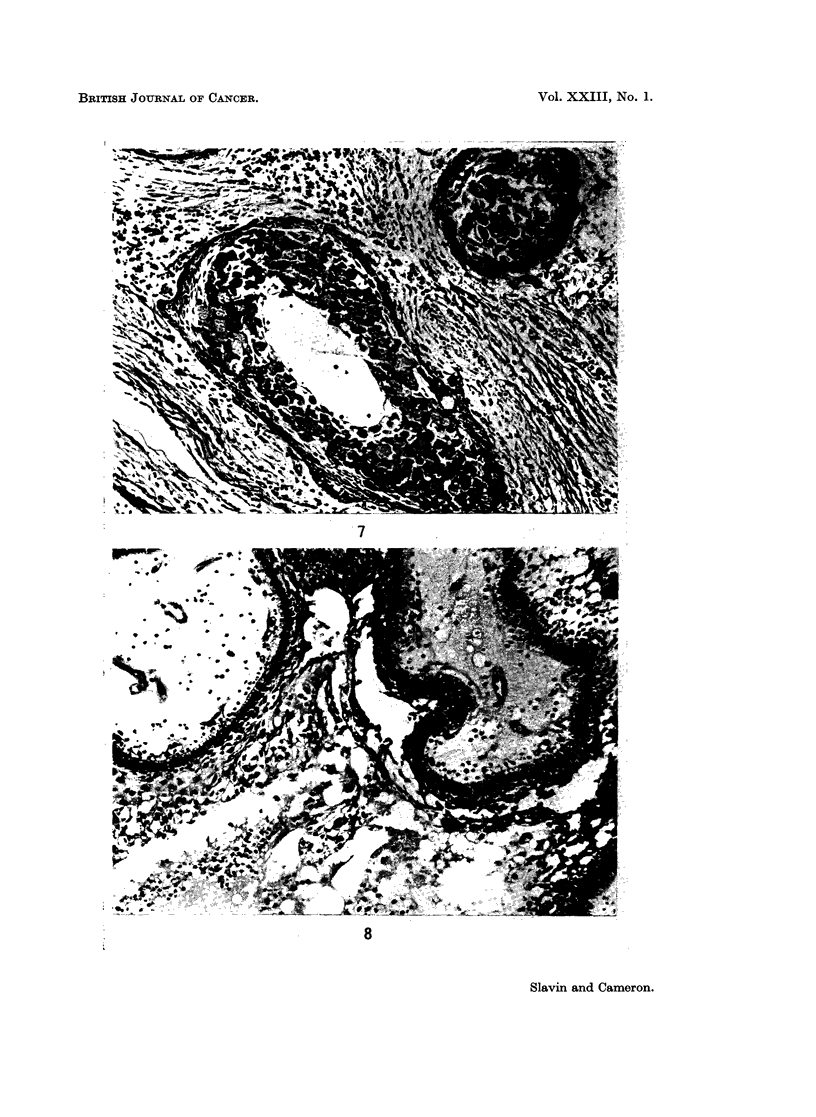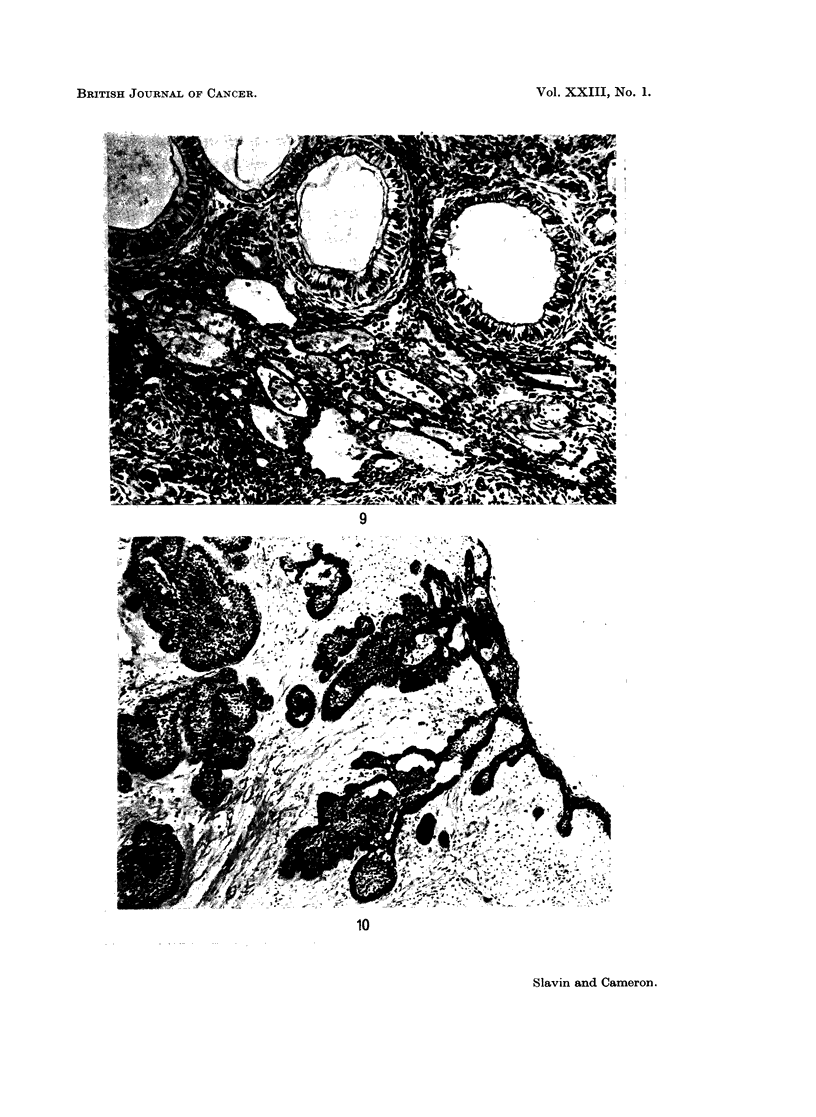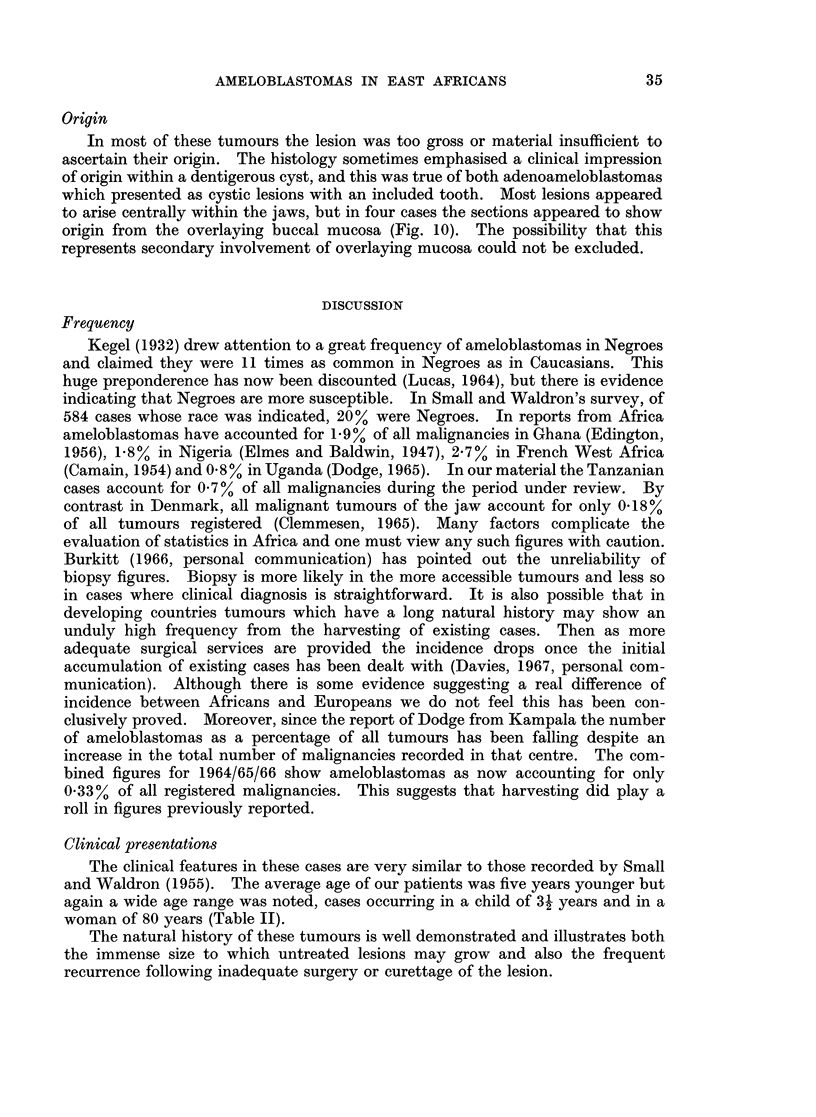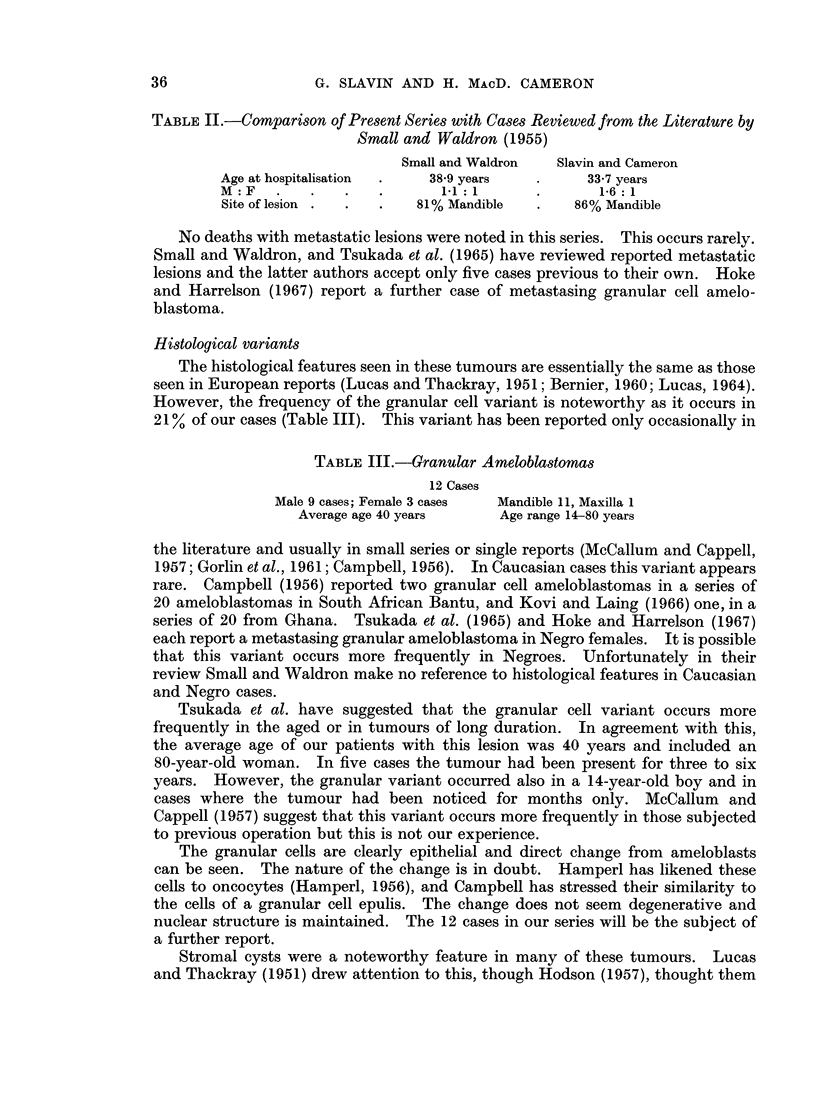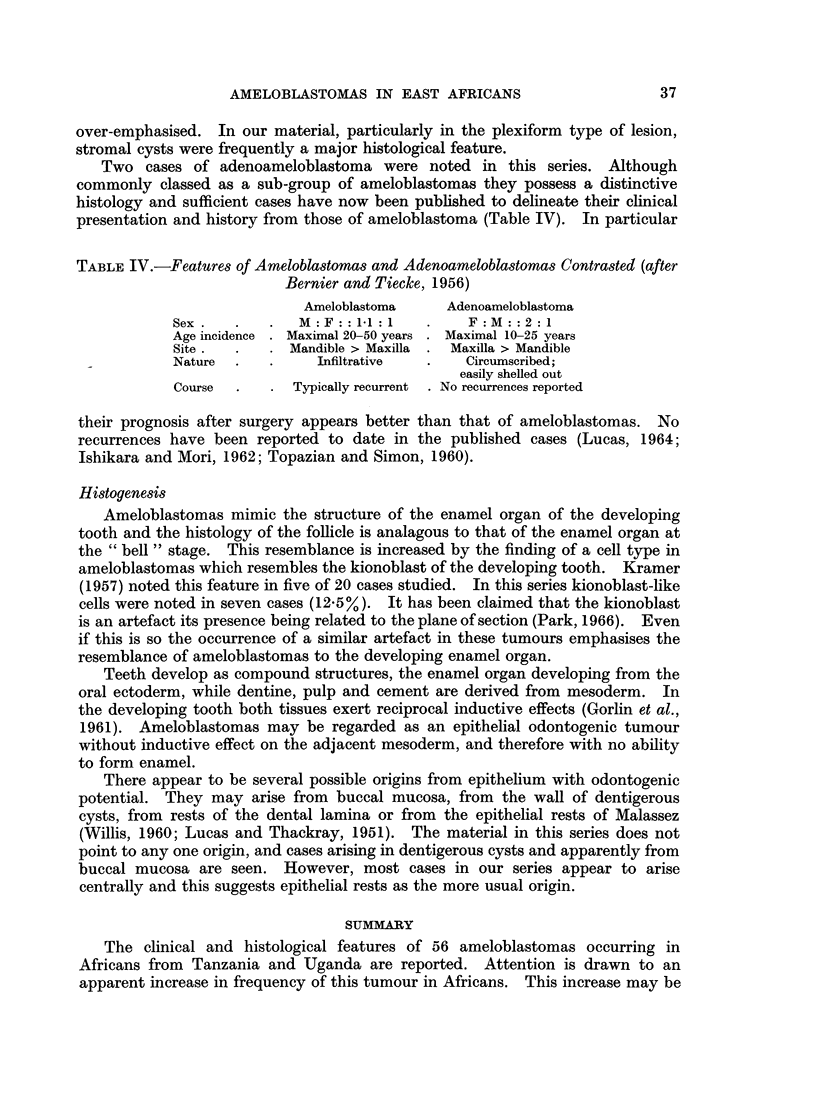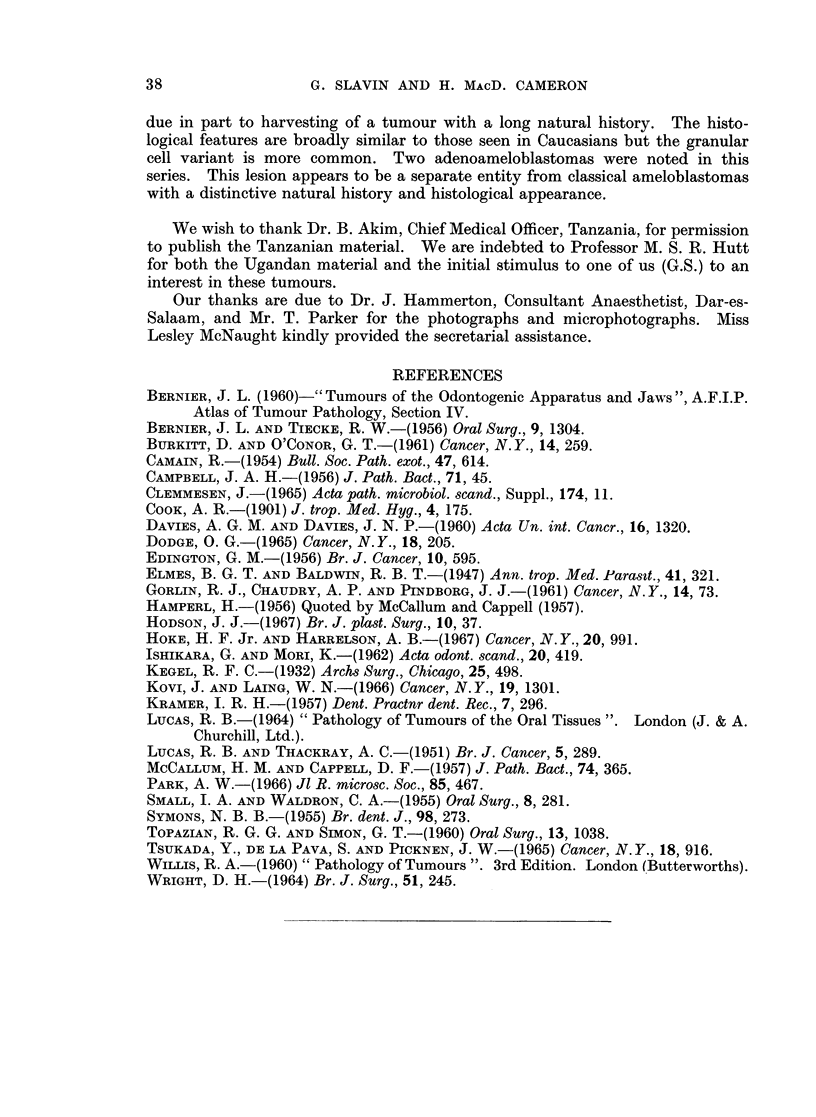# Ameloblastomas in Africans from Tanzania and Uganda. A report of 56 cases.

**DOI:** 10.1038/bjc.1969.5

**Published:** 1969-03

**Authors:** G. Slavin, H. M. Cameron

## Abstract

**Images:**


### AMELOBLASTOMAS IN AFRICANS FROM TANZANIA AND UGANDA

### A Report of 56 Cases

G. SLAVIN* and H. MacD. CAMERON

*University Department of Pathology, Royal Infirmary, Glasgow*

Received for publication October 16, 1968

Sir Alfred Cook drew attention to the great frequency with which jaw tumours are seen in clinical practice in East Africa (Cook, 1901). More recently this frequency of jaw tumours has been underlined in reports from Uganda (Davies and Davies, 1960) and from Ghana (Kovi and Laing, 1966). As has been shown by the work of the Ugandan School (Burkitt and O'Conor, 1961; Wright, 1964), part of this high incidence is due to the lymphomatous syndrome which exists throughout tropical Africa, but there are other jaw tumours which have spectacular clinical presentations and appear to be unduly common. Amongst these, ameloblastomas and benign fibro-osseous lesions of the jaw are prominent (Dodge, 1965). It is the purpose of this paper to review the clinical and histological features of ameloblastomas as they occur in Africans from Tanzania (Mainland Tanganyika) and from Uganda.

### MATERIAL AND METHODS

Material comprises tissue from 56 cases of ameloblastoma—40 of these occurred in Tanzania and are from the records of the Central Pathology Laboratory, Dar-es-Salaam, from 1957–65 inclusive. Sixteen are from the records of the Kampala Tumour Registry for the years 1962–65 inclusive. Eight other cases previously diagnosed in these centres as ameloblastoma were excluded because there was insufficient material left on file to substantiate the diagnosis, and one case was excluded because on review the diagnosis was incorrect.

Each of the cases was examined by H. & E. sections. In selected cases other special stains were done: PAS, Gordon and Sweet's reticulin stain, Masson's trichrome, Van Gieson, and a modified Lendrum's phloxine and tartrazine (Symons, 1955). Clinical details were obtained from biopsy request forms, hospital case notes and information on file in the Kampala Tumour Registry.

### *Clinical findings*

These are summarised in Table I and Fig. 1. Thirty-four cases occurred in males and 21 in females. In one case the sex was not specified. The tumour

Table I.—*Ameloblastomas. Sites of Tumour in 56 Cases*

| | Mandible | Maxilla | Not specified |
|---|---|---|---|
| Right | 11 | 2 | — |
| Left | 10 | 4 | — |
| Bilateral | 4 | — | — |
| Not specified | 23 | — | 2 |
| Total | 48 | 6 | 2 |

* Formerly seconded to the Central Pathology Laboratory, Dar-es-Salaam.

occurred at all ages but was most frequent in the age range 20–50. It occurred in two children aged 3½ and 4½ years. The oldest case was aged 80, and the average age in the 41 cases whose age was known was 33·7 years. Ameloblastoma occurred more frequently in the mandible than the maxilla: 48 cases in the mandible and six in the maxilla. In two cases the site was not specified.

Frequently the initial site of the tumour in the mandible was impossible to discern because of the large size to which the tumour had grown. In those small tumours in which the initial site could be ascertained, the molar region of the

FIG. 1.—Age incidence in 41 cases of ameloblastoma. In 15 cases the adult age was not specified.

mandible appeared to be affected most often. There was no preponderence of either side.

These lesions were often of an immense size causing grotesque disfigurement and great disability (Fig. 2). The largest tumour in this series measured eight inches in greatest diameter and extended from coronoid process to coronoid process. The duration of the tumour preceding hospitalisation was known in 28 cases, and in 23 had been present for less than five years. In one case the tumour had been present for 17 years. The natural history of this lesion following inadequate surgery or curettage was exemplified in many of the histories: one patient had nine operations for recurrences over a period of 17 years, and in six others, single or multiple recurrences were noted.

*Macroscopic appearances*

In the gross specimens examined these tumours were characterised by great distortion of the jaws with expansion of the bone and displacement of the teeth (Fig. 3). They were cystic, classically multilocular, but occasionally unilocular. In the gross specimens we had the opportunity to examine, no purely solid tumours were seen.

*Histology*

The tumours presented two basic histological patterns: follicular (Fig. 4) and plexiform. In the follicular pattern, epithelial islands were set discretely in a connective tissue stroma of variable structure and density; in the plexiform pattern intertwining strands of epithelial tissue ramified through the stroma. Occasionally both patterns occurred in the one tumour, especially if the epithelial islets were large. In this series the follicular pattern was about twice as common as the plexiform.

The follicles resembled the enamel organ of the developing tooth. The outer epithelial layer was tall columnar, cuboidal or less commonly resembled the cells of a basal cell carcinoma. The cytoplasm was clear or eosinophilic and the vesicular nucleus was frequently orientated towards the centre of the follicle and away from the stroma. The resemblance of the outer layer to that of the developing tooth was enhanced in seven cases by the presence of cells with dark elongated nuclei (Fig. 5). These were similar in appearance to the kionoblast cells of the internal enamel epithelium (Symons, 1955). They were best seen using H. & E. preparations, and a modified phloxine and tartrazine did not prove more helpful. It is noteworthy that tumours in which this cell type were noted were follicular in type with tall ameloblasts. The kionoblast-like cells were usually scanty, but in two cases they were numerous.

The central area of the follicles was formed of a loose stellate reticulum. Occasionally between this and the outer ameloblasts a flattened layer of cells resembling normal stratum intermedium was noted. In two cases the central area was formed of more densely spindle areas.

Changes in the central epithelium were common. Cyst formation occurred frequently and appeared to be of two types: either the stellate reticulum gradually faded away, or there was a sharp change between epithelium and cyst. In the latter, the surrounding cells were often flattened and eosinophilic though not squamous, and suggested that the cyst fluid had been under pressure. Moreover, in cysts showing this change altered epithelial cells were often desquamated: these were round and brightly eosinophilic. Some had small pyknotic nuclei, but more frequently there was no nucleus. In the wall of some cysts the change from lining cells to desquamated cells could be followed. The cysts were of variable size and appeared to arise multifocally and enlarge by coalescence.

Squamous metaplasia of the central epithelium was found in 45%. It was largely minor and focal in type but occasionally widespread and with well marked epithelial pearl formation (Fig. 6).

Granular cell change was noted in 21% (Fig. 7). In these the central cells were replaced in part or totally by large eosinophilic granular cells. The nuclei were either central with thick chromatin, or distorted and pushed laterally. They did not show signs of nuclear degeneration. The cells could be seen in transition from the peripheral columnar cells, and occasionally there was replacement of even the outer layer. In tumours showing stromal inflammation stromal macrophages with secondary lipidisation were noted but the appearance of these cells was quite distinct from the granular cells.

Tumours of plexiform pattern showed essentially the same cell types as the follicular, but low cuboidal epithelium was relatively more common.

*Stroma.*—The stroma was variable; in some it was dense and collagenous while in others it had a looser structure. Both extremes were sometimes seen in the same tumour. In one case the connective tissue surrounding the epithelial islets was cellular and formed of young fibroblasts; the appearances were analagous to those seen following induction of odontogenic tissue in the mesoderm of the developing tooth.

In ameloblastomas cysts may form in the stroma as well as in the epithelial follicles and both were found frequently in this series. The two forms were readily distinguished by the arrangement of the stellate reticulum and by the polarity of the ameloblast nuclei at the periphery of the follicle, the nuclei lying towards the stellate reticulum and away from the stroma (Fig. 8). In addition, residual capillaries were found coursing through the stromal cysts.

The series included two examples of the so-called adenoameloblastoma. Both occurred as cystic maxillary tumours in females of 16 and 24 years respectively. Each had previously been diagnosed as an ameloblastoma of atypical structure. They are included here because they are commonly regarded merely as variants of ameloblastoma (Dodge, 1965; Bernier, 1960). They showed the structure typical of adenoameloblastoma with solid areas of gland-like, convoluted and infolded tubular structures alternating with some solid spindle cell areas (Fig. 9). The tall columnar epithelium lining the gland-like areas showed orientation of the nuclei away from the central space which in some was lined by a hyaline eosinophilic membrane. Focal areas of dystrophic calcification were noted. In the stroma, marked capillary dilatation and haemorrhage into stromal cysts was noted. These features were gross enough to simulate the so-called haemangioameloblastoma but no evidence of true capillary proliferation was seen. Stromal vascularity in the other tumours was very variable.

---

### EXPLANATION OF PLATES

Fig. 2.—Bilateral ameloblastoma of mandible producing grotesque enlargement and disfiguration.

Fig. 3.—Mandibulectomy specimen. Whole mandible is expanded, thickened and cystic. Note the gross displacements of the teeth.

Fig. 4.—Ameloblastoma of follicular pattern. The islets are bounded by peripheral columnar cells and centrally there is a loose stellate reticulum with cystic change. H. & E. ×185.

Fig. 5.—Between the cells of the ameloblast layer, cells with elongated dark nuclei and resembling kionoblasts can be seen. H. & E. ×750.

Fig. 6.—Ameloblastoma of follicular pattern with extensive squamous metaplasia. H. & E. ×185.

Fig. 7.—Granular cell ameloblastoma. The inner portion of the epithelial islands are replaced by coarsely granular eosinophilic cells. H. & E. ×185.

Fig. 8.—In the lower portion a large follicular cyst is seen. Two stromal cysts are seen in the upper part of the photograph. Note the contained small blood vessels. H. & E. ×185.

Fig. 9.—Adenoameloblastoma. Gland-like structures formed by tall columnar epithelium and with an internal eosinophilic membrane, are set in a spindle-celled stroma. Note the polarity of the nuclei towards the stroma. H. & E. ×185.

Fig. 10.—Follicular ameloblastoma apparently in continuity with overlying buccal mucosa. H. & E. ×75.

2

Slavin and Cameron.

3

4

5

6

Slavin and Cameron.

7

8

9

10

Slavin and Cameron.

*Origin*

In most of these tumours the lesion was too gross or material insufficient to ascertain their origin. The histology sometimes emphasised a clinical impression of origin within a dentigerous cyst, and this was true of both adenoameloblastomas which presented as cystic lesions with an included tooth. Most lesions appeared to arise centrally within the jaws, but in four cases the sections appeared to show origin from the overlaying buccal mucosa (Fig. 10). The possibility that this represents secondary involvement of overlaying mucosa could not be excluded.

### DISCUSSION

*Frequency*

Kegel (1932) drew attention to a great frequency of ameloblastomas in Negroes and claimed they were 11 times as common in Negroes as in Caucasians. This huge preponderence has now been discounted (Lucas, 1964), but there is evidence indicating that Negroes are more susceptible. In Small and Waldron's survey, of 584 cases whose race was indicated, 20% were Negroes. In reports from Africa ameloblastomas have accounted for 1·9% of all malignancies in Ghana (Edington, 1956), 1·8% in Nigeria (Elmes and Baldwin, 1947), 2·7% in French West Africa (Camain, 1954) and 0·8% in Uganda (Dodge, 1965). In our material the Tanzanian cases account for 0·7% of all malignancies during the period under review. By contrast in Denmark, all malignant tumours of the jaw account for only 0·18% of all tumours registered (Clemmesen, 1965). Many factors complicate the evaluation of statistics in Africa and one must view any such figures with caution. Burkitt (1966, personal communication) has pointed out the unreliability of biopsy figures. Biopsy is more likely in the more accessible tumours and less so in cases where clinical diagnosis is straightforward. It is also possible that in developing countries tumours which have a long natural history may show an unduly high frequency from the harvesting of existing cases. Then as more adequate surgical services are provided the incidence drops once the initial accumulation of existing cases has been dealt with (Davies, 1967, personal communication). Although there is some evidence suggesting a real difference of incidence between Africans and Europeans we do not feel this has been conclusively proved. Moreover, since the report of Dodge from Kampala the number of ameloblastomas as a percentage of all tumours has been falling despite an increase in the total number of malignancies recorded in that centre. The combined figures for 1964/65/66 show ameloblastomas as now accounting for only 0·33% of all registered malignancies. This suggests that harvesting did play a roll in figures previously reported.

*Clinical presentations*

The clinical features in these cases are very similar to those recorded by Small and Waldron (1955). The average age of our patients was five years younger but again a wide age range was noted, cases occurring in a child of 3½ years and in a woman of 80 years (Table II).

The natural history of these tumours is well demonstrated and illustrates both the immense size to which untreated lesions may grow and also the frequent recurrence following inadequate surgery or curettage of the lesion.

TABLE II.—*Comparison of Present Series with Cases Reviewed from the Literature by Small and Waldron* (1955)

| | Small and Waldron | Slavin and Cameron |
|---|---|---|
| Age at hospitalisation | 38·9 years | 33·7 years |
| M : F | 1·1 : 1 | 1·6 : 1 |
| Site of lesion | 81% Mandible | 86% Mandible |

No deaths with metastatic lesions were noted in this series. This occurs rarely. Small and Waldron, and Tsukada *et al.* (1965) have reviewed reported metastatic lesions and the latter authors accept only five cases previous to their own. Hoke and Harrelson (1967) report a further case of metastasing granular cell ameloblastoma.

*Histological variants*

The histological features seen in these tumours are essentially the same as those seen in European reports (Lucas and Thackray, 1951; Bernier, 1960; Lucas, 1964). However, the frequency of the granular cell variant is noteworthy as it occurs in 21% of our cases (Table III). This variant has been reported only occasionally in

TABLE III.—*Granular Ameloblastomas*

12 Cases

| | |
|---|---|
| Male 9 cases; Female 3 cases | Mandible 11, Maxilla 1 |
| Average age 40 years | Age range 14–80 years |

the literature and usually in small series or single reports (McCallum and Cappell, 1957; Gorlin *et al.*, 1961; Campbell, 1956). In Caucasian cases this variant appears rare. Campbell (1956) reported two granular cell ameloblastomas in a series of 20 ameloblastomas in South African Bantu, and Kovi and Laing (1966) one, in a series of 20 from Ghana. Tsukada *et al.* (1965) and Hoke and Harrelson (1967) each report a metastasing granular ameloblastoma in Negro females. It is possible that this variant occurs more frequently in Negroes. Unfortunately in their review Small and Waldron make no reference to histological features in Caucasian and Negro cases.

Tsukada *et al.* have suggested that the granular cell variant occurs more frequently in the aged or in tumours of long duration. In agreement with this, the average age of our patients with this lesion was 40 years and included an 80-year-old woman. In five cases the tumour had been present for three to six years. However, the granular variant occurred also in a 14-year-old boy and in cases where the tumour had been noticed for months only. McCallum and Cappell (1957) suggest that this variant occurs more frequently in those subjected to previous operation but this is not our experience.

The granular cells are clearly epithelial and direct change from ameloblasts can be seen. The nature of the change is in doubt. Hamperl has likened these cells to oncocytes (Hamperl, 1956), and Campbell has stressed their similarity to the cells of a granular cell epulis. The change does not seem degenerative and nuclear structure is maintained. The 12 cases in our series will be the subject of a further report.

Stromal cysts were a noteworthy feature in many of these tumours. Lucas and Thackray (1951) drew attention to this, though Hodson (1957), thought them over-emphasised. In our material, particularly in the plexiform type of lesion, stromal cysts were frequently a major histological feature.

Two cases of adenoameloblastoma were noted in this series. Although commonly classed as a sub-group of ameloblastomas they possess a distinctive histology and sufficient cases have now been published to delineate their clinical presentation and history from those of ameloblastoma (Table IV). In particular

TABLE IV.—*Features of Ameloblastomas and Adenoameloblastomas Contrasted* (*after Bernier and Tiecke*, 1956)

| | Ameloblastoma | Adenoameloblastoma |
|---|---|---|
| Sex | M : F : : 1·1 : 1 | F : M : : 2 : 1 |
| Age incidence | Maximal 20–50 years | Maximal 10–25 years |
| Site | Mandible > Maxilla | Maxilla > Mandible |
| Nature | Infiltrative | Circumscribed; easily shelled out |
| Course | Typically recurrent | No recurrences reported |

their prognosis after surgery appears better than that of ameloblastomas. No recurrences have been reported to date in the published cases (Lucas, 1964; Ishikara and Mori, 1962; Topazian and Simon, 1960).

*Histogenesis*

Ameloblastomas mimic the structure of the enamel organ of the developing tooth and the histology of the follicle is analagous to that of the enamel organ at the " bell " stage. This resemblance is increased by the finding of a cell type in ameloblastomas which resembles the kionoblast of the developing tooth. Kramer (1957) noted this feature in five of 20 cases studied. In this series kionoblast-like cells were noted in seven cases (12·5%). It has been claimed that the kionoblast is an artefact its presence being related to the plane of section (Park, 1966). Even if this is so the occurrence of a similar artefact in these tumours emphasises the resemblance of ameloblastomas to the developing enamel organ.

Teeth develop as compound structures, the enamel organ developing from the oral ectoderm, while dentine, pulp and cement are derived from mesoderm. In the developing tooth both tissues exert reciprocal inductive effects (Gorlin *et al.*, 1961). Ameloblastomas may be regarded as an epithelial odontogenic tumour without inductive effect on the adjacent mesoderm, and therefore with no ability to form enamel.

There appear to be several possible origins from epithelium with odontogenic potential. They may arise from buccal mucosa, from the wall of dentigerous cysts, from rests of the dental lamina or from the epithelial rests of Malassez (Willis, 1960; Lucas and Thackray, 1951). The material in this series does not point to any one origin, and cases arising in dentigerous cysts and apparently from buccal mucosa are seen. However, most cases in our series appear to arise centrally and this suggests epithelial rests as the more usual origin.

### SUMMARY

The clinical and histological features of 56 ameloblastomas occurring in Africans from Tanzania and Uganda are reported. Attention is drawn to an apparent increase in frequency of this tumour in Africans. This increase may be due in part to harvesting of a tumour with a long natural history. The histological features are broadly similar to those seen in Caucasians but the granular cell variant is more common. Two adenoameloblastomas were noted in this series. This lesion appears to be a separate entity from classical ameloblastomas with a distinctive natural history and histological appearance.

We wish to thank Dr. B. Akim, Chief Medical Officer, Tanzania, for permission to publish the Tanzanian material. We are indebted to Professor M. S. R. Hutt for both the Ugandan material and the initial stimulus to one of us (G.S.) to an interest in these tumours.

Our thanks are due to Dr. J. Hammerton, Consultant Anaesthetist, Dar-es-Salaam, and Mr. T. Parker for the photographs and microphotographs. Miss Lesley McNaught kindly provided the secretarial assistance.

### REFERENCES

Bernier, J. L. (1960)—"Tumours of the Odontogenic Apparatus and Jaws", A.F.I.P. Atlas of Tumour Pathology, Section IV.
Bernier, J. L. and Tiecke, R. W.—(1956) *Oral Surg.*, **9**, 1304.
Burkitt, D. and O'Conor, G. T.—(1961) *Cancer, N.Y.*, **14**, 259.
Camain, R.—(1954) *Bull. Soc. Path. exot.*, **47**, 614.
Campbell, J. A. H.—(1956) *J. Path. Bact.*, **71**, 45.
Clemmesen, J.—(1965) *Acta path. microbiol. scand.*, Suppl., **174**, 11.
Cook, A. R.—(1901) *J. trop. Med. Hyg.*, **4**, 175.
Davies, A. G. M. and Davies, J. N. P.—(1960) *Acta Un. int. Cancr.*, **16**, 1320.
Dodge, O. G.—(1965) *Cancer, N.Y.*, **18**, 205.
Edington, G. M.—(1956) *Br. J. Cancer*, **10**, 595.
Elmes, B. G. T. and Baldwin, R. B. T.—(1947) *Ann. trop. Med. Parasit.*, **41**, 321.
Gorlin, R. J., Chaudry, A. P. and Pindborg, J. J.—(1961) *Cancer, N.Y.*, **14**, 73.
Hamperl, H.—(1956) Quoted by McCallum and Cappell (1957).
Hodson, J. J.—(1967) *Br. J. plast. Surg.*, **10**, 37.
Hoke, H. F. Jr. and Harrelson, A. B.—(1967) *Cancer, N.Y.*, **20**, 991.
Ishikara, G. and Mori, K.—(1962) *Acta odont. scand.*, **20**, 419.
Kegel, R. F. C.—(1932) *Archs Surg., Chicago*, **25**, 498.
Kovi, J. and Laing, W. N.—(1966) *Cancer, N.Y.*, **19**, 1301.
Kramer, I. R. H.—(1957) *Dent. Practnr dent. Rec.*, **7**, 296.
Lucas, R. B.—(1964) " Pathology of Tumours of the Oral Tissues ". London (J. & A. Churchill, Ltd.).
Lucas, R. B. and Thackray, A. C.—(1951) *Br. J. Cancer*, **5**, 289.
McCallum, H. M. and Cappell, D. F.—(1957) *J. Path. Bact.*, **74**, 365.
Park, A. W.—(1966) *Jl R. microsc. Soc.*, **85**, 467.
Small, I. A. and Waldron, C. A.—(1955) *Oral Surg.*, **8**, 281.
Symons, N. B. B.—(1955) *Br. dent. J.*, **98**, 273.
Topazian, R. G. G. and Simon, G. T.—(1960) *Oral Surg.*, **13**, 1038.
Tsukada, Y., de la Pava, S. and Picknen, J. W.—(1965) *Cancer, N.Y.*, **18**, 916.
Willis, R. A.—(1960) " Pathology of Tumours ". 3rd Edition. London (Butterworths).
Wright, D. H.—(1964) *Br. J. Surg.*, **51**, 245.